# Hyperconnectivity of the lateral amygdala in long-term methamphetamine abstainers negatively correlated with withdrawal duration

**DOI:** 10.3389/fphar.2023.1138704

**Published:** 2023-11-10

**Authors:** Yifan Li, Xuhao Wang, Shucai Huang, Qiuping Huang, Ru Yang, Zhenjiang Liao, Xinxin Chen, Shuhong Lin, Yongyan Shi, Chenhan Wang, Ying Tang, Jingyue Hao, Jie Yang, Hongxian Shen

**Affiliations:** ^1^ Department of Psychiatry, and National Clinical Research Center for Mental Disorders, The Second Xiangya Hospital of Central South University, Changsha, Hunan, China; ^2^ Department of Psychiatry, The Fourth People’s Hospital of Wuhu, Wuhu, Anhui, China; ^3^ Department of Applied Psychology, School of Humanities and Management, Hunan University of Chinese Medicine, Changsha, China; ^4^ Department of Radiology, The Second Xiangya Hospital of Central South University, Changsha, Hunan, China; ^5^ The First Clinical Medical College of Lanzhou University, Lanzhou, China

**Keywords:** long-term withdrawal, functional connectivity, amygdala, occipital gyrus, temporal gyrus

## Abstract

**Introduction:** Several studies have reported structural and functional abnormalities of the amygdala caused by methamphetamine addiction. However, it is unknown whether abnormalities in amygdala function persist in long-term methamphetamine abstainers.

**Methods:** In this study, 38 long-term male methamphetamine abstainers (>12 months) and 40 demographically matched male healthy controls (HCs) were recruited. Considering the heterogeneous nature of the amygdala structure and function, we chose 4 amygdala subregions (i.e., left lateral, left medial, right lateral, and right medial) as regions of interest (ROI) and compared the ROI-based resting-state functional connectivity (FC) at the whole-brain voxel-wise between the two groups. We explored the relationship between the detected abnormal connectivity, methamphetamine use factors, and the duration of withdrawal using correlation analyses. We also examined the effect of methamphetamine use factors, months of withdrawal, and sociodemographic data on detected abnormal connectivity through multiple linear regressions.

**Results:** Compared with HCs, long-term methamphetamine abstainers showed significant hyperconnectivity between the left lateral amygdala and a continuous area extending to the left inferior/middle occipital gyrus and left middle/superior temporal gyrus. Abnormal connections negatively correlated with methamphetamine withdrawal time (r = −0.85, *p* < 0.001). The linear regression model further demonstrated that the months of withdrawal could identify the abnormal connectivity (β_adj_ = −0.86, 95%CI: −1.06 to −0.65, *p* < 0.001).

**Discussion:** The use of methamphetamine can impair the neural sensory system, including the visual and auditory systems, but this abnormal connectivity can gradually recover after prolonged withdrawal of methamphetamine. From a neuroimaging perspective, our results suggest that withdrawal is an effective treatment for methamphetamine.

## 1 Introduction

Methamphetamine is a common amphetamine-type stimulant that excites the central nervous system. Methamphetamine abuse leads to cognitive impairment, increased impulsiveness, and aggression (Vocci, 2008; Hart et al., 2012; Cservenka and Ray, 2017; Wang et al., 2017), as well as severe psychotic symptoms, including visual hallucinations, auditory hallucinations, and persecution delusions (Grant et al., 2012; Yang et al., 2021; Edinoff et al., 2022). Additionally, the repeated use of methamphetamine can affect the hypothalamic-pituitary-adrenal (HPA) axis, which can result in a state of anxiety and depression (Li et al., 2013; Zuloaga et al., 2015), and even an increased risk of suicide (Berardelli et al., 2020). According to the *World Drug Report 2022*, in the past decade, the number of amphetamine users worldwide has been steadily increasing, surpassing traditional drugs such as heroin and ketamine. Methamphetamine is ranked as one of the most abused drugs worldwide. In China, methamphetamine is the most abused drug, with 57.2% of its users using it (World Drug Report, 2022). Currently, there is no effective treatment for methamphetamine use disorder (Shoptaw et al., 2009; Acheson et al., 2022), although withdrawal treatment has a certain effect (McCann et al., 2008; Iudicello et al., 2010; Zorick et al., 2010; Proebstl et al., 2019; Paulus and Stewart, 2020; Liu et al., 2021). Withdrawal treatment is one of the primary methods used by the Chinese government to control the use of methamphetamine (Shanghai Drug Rehabilitation Administration Subject Group, 2023).

Withdrawal treatment is a detoxification method that interrupts drug use and manages withdrawal symptoms. It can improve impulse inhibition (Liu et al., 2021), cognitive deficits (McCann et al., 2008; Iudicello et al., 2010; Proebstl et al., 2019), and psychotic symptoms (Zorick et al., 2010). However, this treatment also has shortcomings, specifically, abrupt cessation of methamphetamine use can lead to withdrawal syndrome including anxiety, anhedonia (Iudicello et al., 2010; Su et al., 2017; Proebstl et al., 2019; Acheson et al., 2022), and may even experience relapse behavior (Zuloaga et al., 2015) and a higher risk of treatment resistance (Serafini et al., 2017). Even after a short-term withdrawal, methamphetamine users may still have abnormal social functions, such as low social acceptance, violent behavior, and affective symptoms (Kim et al., 2011). With long-term withdrawal treatment, some of these symptoms gradually resolve, but others continue to develop into persistent symptoms, such as insomnia and anxiety (Peck et al., 2005; McCann et al., 2008; Iudicello et al., 2010; Proebstl et al., 2019). Assessing the neural mechanisms of long-term methamphetamine abstainers can help understand 1) the efficacy of withdrawal and 2) the long-term effects of methamphetamine on the neural system. However, few studies have shed light on the neural mechanisms of long-term abstaining (beyond the first 6 months of withdrawal).

Together with the nucleus accumbens, ventral tegmental area, thalamus, hippocampus, and medial prefrontal cortex, the amygdala forms a reward circuit in the brain that plays an important role in substance addiction and abuse (Huang et al., 2018; Yang et al., 2022). Previous studies reported that methamphetamine can cause structural and functional abnormalities in the amygdala (Dean et al., 2014; Okita et al., 2016). Methamphetamine users had reduced gray matter volume (Orikabe et al., 2011) and increased activation in the amygdala, resulting in poor regulation of emotion, increased aggression, and impulsivity, which may further exacerbate addictive behaviors in methamphetamine users (Payer et al., 2011). The amygdala is a heterogeneous structure that is further subdivided into lateral and medial parts. The lateral amygdala is associated with fear extinction and reward processes (Zhang et al., 2020; Choi et al., 2021), whereas the medial amygdala is associated with various social behaviors, stress responses, and aggression (Haller, 2018; Raam and Hong, 2021). Therefore, dividing the amygdala into multiple subregions could help us better understand its different roles in addiction.

Although several neuroimaging studies have investigated the neural mechanisms of acute methamphetamine users, few have investigated the neural mechanisms of long-term methamphetamine abstainers. Inspired by research reported the structural and functional abnormalities in the amygdala of methamphetamine addictions, and taking into account the heterogeneity nature of amygdala structure and function (Fan et al., 2016; Kang et al., 2017), we speculate that individuals who are long-term abstinent from methamphetamine may continue to exhibit functional abnormalities in amygdala, but the abnormalities of amygdala subregions may vary. This study aims to explore the resting-state functional connectivity (FC) between different amygdala subregions with other brain regions in individuals who long-term abstinent from methamphetamine, and to examine the relationship between these abnormal connections with withdrawal time, frequency of methamphetamine use, dosage, and other related factors.

## 2 Materials and methods

### 2.1 Participants

This was a retrospective case-control study. The participants included 47 male long-term methamphetamine abstainers and 44 age-matched healthy male volunteers. Long-term methamphetamine abstainers were obtained from the Pingtang Isolation and Compulsory Drug Rehabilitation Center of Hunan Province, China. The inclusion criterion for the methamphetamine withdrawal group was age: 18–45 years; Han nationality; right-handed; meeting the Diagnostic and Statistical Manual of Mental Disorders (DSM-IV) criteria for methamphetamine dependence; and using methamphetamine twice weekly for >2 years; duration of abstinence >12 months.

The exclusion criteria were illiteracy; lifetime diagnosis of substance dependence other than methamphetamine; Past serious I-axis medical, neurological, or psychotic disorders; current use of psychotropic or intravenous drugs; Patient Health Questionnaire-9 (PHQ-9) item (Spitzer, 1999; Kroenke et al., 2001) scores >5 or suicidal ideation, claustrophobia, learning disability, or central nervous system illness; traumatic brain injury with fracture or loss of consciousness for at least 10 min; and other contraindications for magnetic resonance imaging (MRI). The healthy controls (HCs) comprised local residents, male, 18–45 years old, Han nationality, and right-handed. Current or former methamphetamine users who met the DSM-IV criteria or had serious medical and/or neurological problems were excluded. All subjects were asked to abstain from alcohol, nicotine, or other potentially psychoactive substances for at least 48 h prior to the study and HCs were additionally required to have a score of less than four on the Alcohol Use Disorders Identification Test-Concise (AUDIT-C) (Bush, 1998). All research procedures were conducted in accordance with the ethical norms of the 1975 Declaration of Helsinki and were reviewed and approved by the Institutional Review Committee of Second Xiangya Hospital of Central South University. All participants were fully informed of the study procedures and provided signed informed consent. This study adhered to the Strengthening the Reporting of Observational Studies in Epidemiology (STROBE) statement.

### 2.2 Measurements

Three days before the start of the study, the urine of the abstainers was tested to assess whether they had recently used drugs. Self-report questionnaires were collected on the day the study began or the day before. These questionnaires were used to collect demographic information, methamphetamine use information, and months of abstinence. Demographic information included age, marital status, years of education, monthly income before entering rehabilitation, and whether they smoked and drank, while information on methamphetamine use included frequency of methamphetamine use in the past year and month before enrollment, monthly methamphetamine doses before entering rehabilitation, age of first use, and months of methamphetamine-use. The Edinburgh Handedness Scale was used to determine the dominant hand of each participant.

### 2.3 Scanning parameters of functional MRI

Scans were performed using a Tesla Siemens 3.0 scanner (Allegra; Siemens Medical Systems, Erlangen, Germany) with a standard head coil. Whole-brain echo planar image from functional MRI (fMRI) scan with T2-weighted gradient echo sequence, repetition time (TR) = 2000 ms, echo time = 30 ms, flip angle = 80°, field of view (FOV) = 220 mm × 220 mm, voxel size = 3.4 mm × 3.4 mm × 4.0 mm; slice thickness = 4 mm; gap = 1 mm; matrix = 64 × 64; number of slices = 36, volume = 225. The total fMRI scan duration was 450 s. Earplugs and pillows were placed around the head to isolate and control head movement, respectively.

### 2.4 fMRI data preprocessing

The MATLAB-based SPM12 toolkit (SPM12 https://www.fil.ion.ucl.ac.uk/spm/) and DPABI (http://rfmri.org/dpabi) were used to preprocess the data. The steps included 1) image format conversion, converting DICOM format to NII format; 2) removal of the first 10 images to ensure the stability of the magnetic field and to give subjects time to adapt; 3) time layer correction, selecting the layer acquired at the middle time point as the reference layer, and correcting the other layers to make them consistent with the acquisition time of the reference layer; 4) head movement correction, remove of subjects whose head movements greater than 2 mm and 2°, which included four HCs and nine methamphetamine abstainers; 5) spatial normalization, aligning all images to Montreal Neurological Institute (MNI) space by Diffeomorphic Anatomical Registration Through Exponentiated Lie Algebra (DARTEL); 6) covariate removal, including 24 head motion parameters, cerebral white matter, cerebrospinal fluid and whole brain noise signals; 7) removal of linear drift; 8) smoothing, with a Gaussian smoothing kernel half-height width of 8 mm; 9) filtering, leaving data in the 0.01–0.08 Hz interval to reduce low frequency drift and physiological noise.

### 2.5 Functional connectivity analyses

Four amygdala subregions (left lateral, left medial, right lateral, and right medial) were selected as regions of interest (ROI). Time series correlations between the four ROIs and other regions in the whole brain were calculated voxel-wise, and Fisher’s Z transformation was performed to obtain normalized FC values.

### 2.6 Statistical analyses

General demographic and clinical data were analyzed using SPSS 23.0, with a two-sided test and a significance level of *p* < 0.05. The DPABI was applied as an independent samples *t*-test for FC values in the two groups, with age and years of education as covariates. The statistical significance level was set to Gaussian random field (GRF) correction with a voxel *p* < 0.05, and areas with a corrected cluster *p* < 0.05.

### 2.7 Linear regression analyses

Pearson’s correlations were used to examine the relationship between FC strength and methamphetamine use (including months of use, dose, frequency of use in the previous year and last month, age at first methamphetamine use, and months of withdrawal). Multifactorial linear regression equations were used to explore the independent risk factors for changes in brain function. Variance inflation factors (VIF) were used to determine multicollinearity between the independent variables, with VIF > 5 indicating multicollinearity and VIF > 10 indicating severe multicollinearity. If VIF > 10, one of the two variables with strong covariance was removed.

## 3 Results

### 3.1 General demographic and drug use characteristics

After data quality analysis, there were 38 people in the long-term methamphetamine abstainer group and 40 in the HC group. No group-related differences in age, educational level, monthly income, smoking status, and drinking status were observed. Regarding marital status, the ratio of unmarried to divorced individuals was significantly higher in the methamphetamine withdrawal group than in the control group (*χ2* = 26.57, *p* < 0.001), and the demographic data is provided in Table 1.

**TABLE 1:** Demographics in methamphetamine long-term abstainers and healthy control group.

	Healthy control group	Methamphetamine long-term abstainers	*t/χ2*	*p*
	(*n* = 40)	(*n* = 38)		
Age	34.38 ± 7.58	32.55 ± 6.69	1.12	0.26
Years of education	9.65 ± 2.34	8.84 ± 2.18	1.58	0.12
Monthly income before entering rehab (¥)	4,090.00 ± 2,460.85	4,257.89 ± 3,287.21	−0.26	0.80
Marital Status				
Married	32 (80.0%)	10 (26.3%)	26.57	<0.001
Unmarried	8 (20.0%)	23 (60.5%)		
Divorce	0	5 (13.2%)		
Number of smokers^a^ (percentage)	38 (95.0%)	37 (97.4%)	0.30	0.59
Number of people who ever used alcohol^b^ (percentage)	7 (17.5%)	14 (36.8%)	3.71	0.05

Note: a: For the purposes of this study, “smokers” were defined as people who had ever used cigarettes, healthy controls were asked to have not used cigarettes for 48 h, while abstainers had no access to cigarettes. b: For the purposes of this study, healthy controls were asked to have a score of less than 4 on the Alcohol Use Disorders Identification Test-Concise and to refrain from drinking alcohol for 48 h, while abstainers had no access to alcohol.

In the methamphetamine withdrawal group, the average first-use age was 25.92 ± 6.89 years, the average use time was 59.92 ± 32.42 months, the average use frequencies in the last year and last month were 2.08 ± 1.08 times and 2.00 ± 1.23 times, respectively. The average dose at the last month was 24.58 ± 25.12 g, indicating that the participants recruited in this study had severe methamphetamine abuse before entering the drug rehabilitation center. The average number of months of withdrawal after entering the drug rehabilitation center was 19.16 ± 2.55 months. Please refer to Table 2 for the detailed usage of methamphetamine.

**TABLE 2:** Details of methamphetamine use characteristics and withdrawal months.

	Methamphetamine long-term abstainers (*n* = 38)	Range
Age of the subjects at the first use	25.92 ± 6.89	15–39
Months of methamphetamine use	59.92 ± 32.42	22–190
Frequency in the last year^a^	2.08 ± 1.08	1–4
Frequency in the last month^a^	2.00 ± 1.23	1–4
Dose per month (g)	24.58 ± 25.12	1–90
months of withdrawal	19.16 ± 2.55	14–25

Note: a: “Last Year” or “Last Month” means the last year or month before the methamphetamine long-term abstainers entered Pingtang Isolation and Compulsory Drug Rehabilitation Center.

### 3.2 Functional connectivity analyses

**FIGURE 1:**
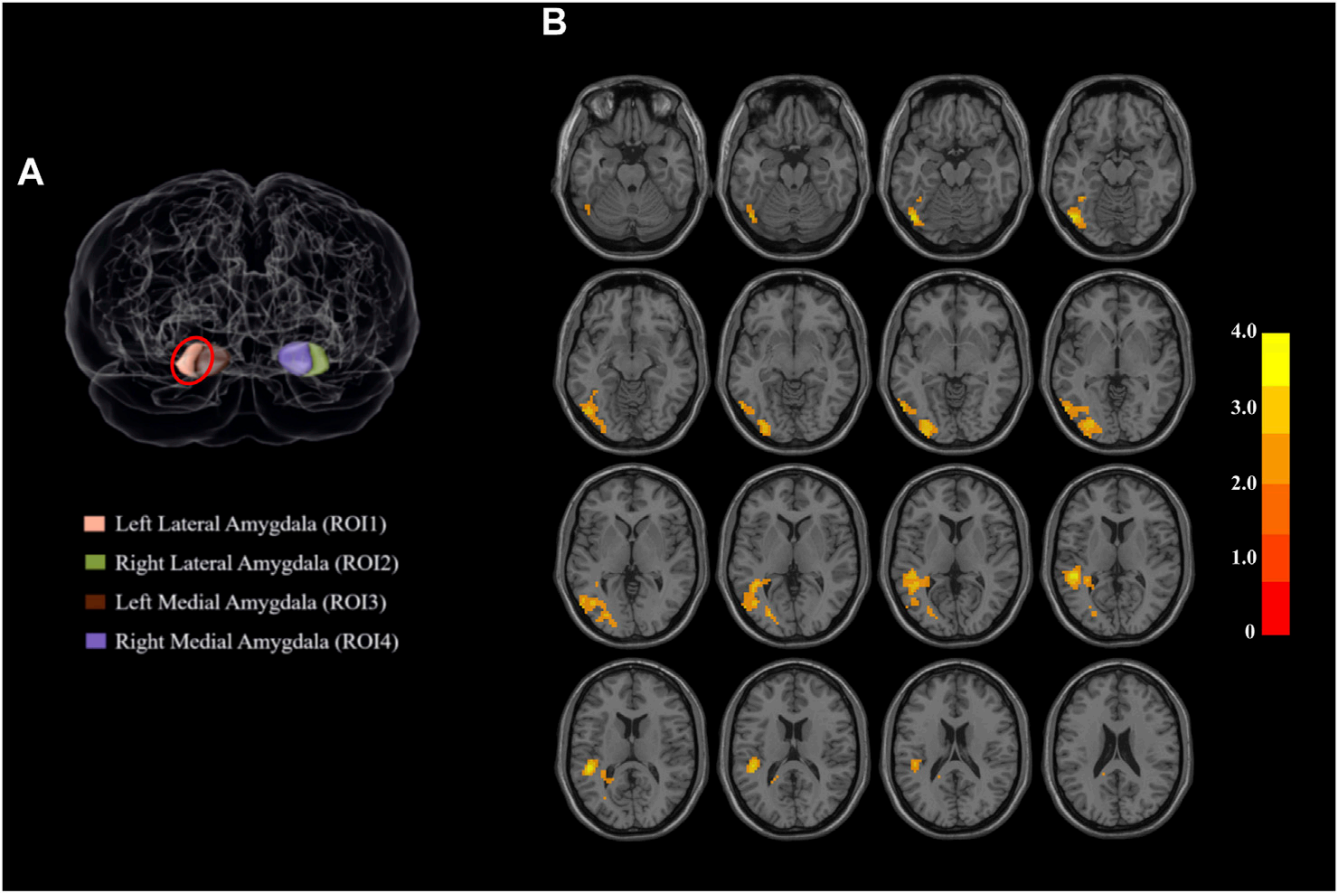
Regions of interest and the regions that differed significantly between the two groups. **(A)** Amygdala subdivisions from a dorsal view of the brain. The amygdala is segmented into four subregions based on the human Brainnetome Atlas. When the left lateral amygdala was used as the region of interest, the functional connectivity of the two populations showed a difference between groups. Red circle: left lateral amygdala. **(B)** Functional connectivity of parts of the superior temporal gyrus with the left lateral amygdala shows group-related differences. Abbreviations: ROI, region of interest.

As presented in Table 3; Figure 1, the methamphetamine abstainers group showed hyperconnectivity of the left lateral amygdala with a continuous area that extended to the left inferior occipital gyrus, left middle occipital gyrus, left middle temporal gyrus, and left superior temporal gyrus compared with HCs. However, no abnormal connectivity was detected in the methamphetamine abstainers group when the other 3 amygdala subregions were used as ROI.

**TABLE 3:** Brain regions showing group-related difference in the functional connectivity with the left lateral amygdala.

Cerebral hemisphere	Cluster size (voxels)	*t*	MNI^a^ coordinate			Overlap (%)	Label include
			x	y	z		
Left	633	3.88	−48	−36	9	12	Superior temporal gyrus
						18	Middle occipital gyrus
						17	Middle temporal gyrus
						10	Inferior occipital gyrus
						6	Inferior temporal gyrus
						4	Fusiform gyrus
						3	Rolandic operculum
						2	Cerebellum_Crus1

Note: a: MNI, Coordinate = Montreal Neurological Institute Coordinate.

### 3.3 Linear regression analyses

**FIGURE 2:**
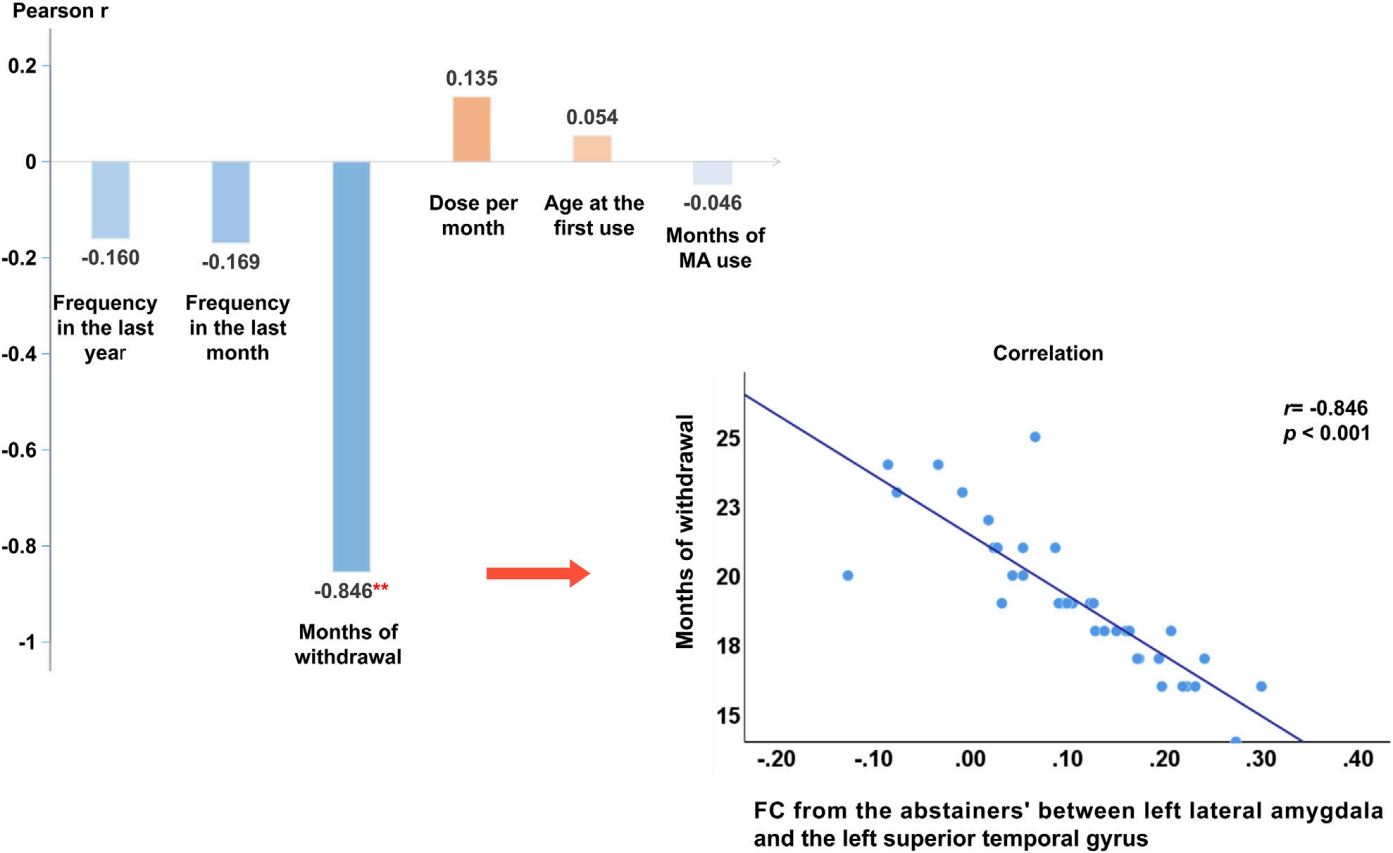
Pearson’s correlation results and Scatter plot of functional connectivity strength between the detected functional connectivity with months of withdrawal. Note: **: At the α = 0.01 level (two-tailed), the correlation is significant. Months of withdrawal was negatively correlated with the strength of the functional connectivity (r = −0.85, *p* < 0.001), other variables were not found to be significantly associated with functional connectivity strength. Abbreviations: MA, Methamphetamine.

We extracted the average connectivity between the left lateral amygdala and the detected area and conducted a Pearson correlation to examine the relationship between this abnormal connectivity and methamphetamine use characteristics and months of withdrawal. Abnormal connectivity was negatively correlated with the number of months since withdrawal (*r* = −0.85, *p* < 0.001). After incorporating methamphetamine use characteristics, months of withdrawal, and sociodemographic data into the multiple linear regression equation, months of withdrawal independently identified abnormal functional connectivity (β_adj_ = −0.86, 95%CI: −1.06 to −0.65, *p* < 0.001). Please refer to Figure 2 and Table 4 for further details.

**TABLE 4:** Multiple linear regression results.

Variables	*β*	*β*_*adj*_^*a*^	*t*	*p*	95% CI (β_adj_)		Collinearity statistics	
					Low limit	High limit	Volume	Variance inflation factors
Age	−0.005	−0.313	−1.15	0.26	−0.873	0.247	0.102	9.815
Marital Status	0.005	0.029	0.30	0.76	−0.170	0.229	0.802	1.247
Years of education	−0.008	−0.181	−1.87	0.07	−0.381	0.019	0.801	1.249
Monthly income	1.73 × 10^−6^	0.057	0.54	0.60	−0.161	0.274	0.675	1.482
Smoking	0.001	0.002	0.02	0.98	−0.185	0.190	0.908	1.101
Drinking	−0.010	−0.051	−0.53	0.60	−0.249	0.147	0.815	1.226
Frequency in the last year^b^	−0.008	−0.083	−0.61	0.55	−0.366	0.199	0.400	2.503
Frequency in the last month^b^	0.009	0.108	0.70	0.49	−0.212	0.429	0.311	3.216
Dose per month	−8.11 × 10^−5^	−0.020	−0.15	0.88	−0.295	0.254	0.425	2.355
Age of the subjects at the first use	0.004	0.304	1.18	0.25	−0.227	0.836	0.113	8.845
Months of methamphetamine use	0.001	−0.096	−0.95	0.35	−0.305	0.113	0.733	1.365
Months of withdrawal	−0.034	−0.858	−8.50	<0.001*	−1.065	−0.650	0.739	1.353

Note: a: β_adj_ = Adjusted normalization factor; b: “Last Year” or “Last Month” means the last year or month before the methamphetamine withdrawal population entered Pingtang Isolation and Compulsory Drug Rehabilitation Center. Dependent variable: the functional connectivity between the left lateral amygdala and the left superior temporal gyrus.

## 4 Discussion

By analyzing amygdala-based resting-state FC in long-term methamphetamine abstainers, the current study has reported several following findings. First, we observed that in terms of marital status, the proportion of unmarried and divorced was significantly higher in the long-term methamphetamine abstainers than that in the HCs. What’s more, compared with the HCs, long-term methamphetamine abstainers showed hyperconnectivity between the left lateral amygdala with a continuous area extending to the left inferior/middle occipital gyrus, and the left middle/superior temporal gyrus. Finally, Pearson correlation analysis and multivariate linear regression revealed that months of withdrawal were negatively correlated with the detected FC (r = −0.85, *p* < 0.001), which is also an independent predictor of abnormal FC (β_adj_ = −0.86, 95% CI: −1.06 to −0.65, *p* < 0.001).

In this study, there were no significant group-related differences in age, educational level, monthly income, smoking status, or alcohol consumption. In terms of marital status, the ratio of unmarried to divorced long-term methamphetamine abstainers was significantly higher than that of the HCs (*χ2* = 26.57, *p* < 0.001). This suggests that it is more difficult for methamphetamine users to maintain a healthy family life with others compared to non-users. A previous study reported that methamphetamine users were mostly unmarried (48.57%), divorced, separated, or widowed (15.14%) (Su et al., 2018). Furthermore, long-term methamphetamine abstainers included in this study were recruited from isolated drug rehabilitation centers. They seldom have contact with the outside world or participate in social activities; therefore, it is difficult for them to find suitable marriages. Stigma and discrimination against methamphetamine users are also common, which can create negative and derogatory impressions, resulting in less contact between methamphetamine users and the outside world (Deen et al., 2021).

In the current study, compared to HCs, long-term methamphetamine abstainers showed hyperconnectivity between the left lateral amygdala and left inferior/middle occipital gyrus. These areas are located in the occipital lobe, which is primarily responsible for processing visual, motor, and language information. Sato et al. (2017) reported that both the inferior occipital gyrus and amygdala are involved in facial recognition (Sato et al., 2017). Methamphetamine use may cause abnormalities in the visual system. Previous studies have reported that psychotic symptoms, such as visual hallucinations, may occur with continued methamphetamine use (Onitsuka et al., 2007; Grant et al., 2012). These symptoms appear during methamphetamine use and persist during the withdrawal period (Akiyama et al., 2011; Potvin et al., 2018). Van Hedger et al. have reported that methamphetamine users showed increased activation of primary visual centers in response to low “non-straight edges” (NSE) or less complex stimuli during an NSE visual stimulation task (Van Hedger et al., 2019). Based on these findings, we suggest that methamphetamine has long-term effects on the visual system.

Increased FC between the left lateral amygdala and left inferior/middle occipital gyrus may also be associated with increased sexual impulsiveness in long-term methamphetamine abstainers. Numerous studies have demonstrated that methamphetamine use enhances sexual assertiveness and reduces sexual inhibition (Giorgetti et al., 2017; Chen et al., 2020). Activities in the amygdala and occipital lobe are associated with sexual impulsivity (Gola et al., 2015; Huang et al., 2018; Iovino et al., 2019). Previous studies have reported increased activity in the amygdala when primitive impulses were enhanced (Gola et al., 2015; Iovino et al., 2019). However, the sexual activity of methamphetamine users confined to drug rehabilitation centers is inhibited (Bismpas et al., 2020), and sexual impulses during withdrawal treatment are increased (Semple et al., 2005; Jones et al., 2016). Our previous study also showed that long-term methamphetamine abstainers showed increased activity in the occipital lobe when exposed to pornographic cues (Huang et al., 2018).

Long-term methamphetamine abstainers showed hyperconnectivity between the left lateral amygdala and left middle/superior temporal gyrus compared to HCs. Previous studies have demonstrated a functional crossover between the amygdala and the voice-sensitive auditory cortex, which is located along the superior temporal gyrus and sulcus bilaterally and is often associated with auditory and emotional processing (Pernet et al., 2015; Pannese et al., 2016). The lateral amygdala is an important subregion that receives auditory input (Yang et al., 2022), When auditory information is delivered to the brain, both the amygdala and auditory cortex can process and decode emotional information in auditory information to discriminate emotions in sounds (Ousdal et al., 2014; Pannese et al., 2016). The current study found increased FC between the amygdala and superior temporal gyrus in long-term methamphetamine withdrawal, indicating that some brain regions involved in the auditory circuit are affected by methamphetamine. Furthermore, the middle temporal gyrus receives information from the occipital lobe for visual processing, facial recognition, and emotional recognition (Pourtois et al., 2005). Combined with the present finding of abnormal FC between the amygdala and occipital lobe, we speculated that methamphetamine use would cause some degree of effects on some brain regions involved in emotion regulation and sensory recognition and that these adverse effects could persist in the long term.

In the current study, abnormal FC in long-term methamphetamine abstainers was observed only when the left lateral amygdala was used as the ROI. Previous studies have reported imbalanced functions in different parts of the amygdala; for example, the left amygdala is more recruited in emotional processing than the right amygdala (Peng et al., 2020); whereas the lateral and medial amygdala are associated with terror acquisition, stress response, and aggression, respectively (Haller, 2018; Raam and Hong, 2021). Consistent with the results of the current study, Hayes et al. (2003) reported that the basolateral amygdala complex mediates drug-seeking behaviors in cocaine-dependent rats (Hayes et al., 2003). Furthermore, Li et al. have reported that the lateral amygdala is associated with the reward processes (Li et al., 2018). We speculate that abnormalities in the left lateral amygdala may be more prominent in addicted brains and call for future relevant research focusing on this amygdala subregion.

This study observed that withdrawal duration could independently identify abnormal hyperconnectivity after excluding the effects of other factors. Notably, a more lenient multiple-comparison correction method was used to derive the results. These findings suggest that methamphetamine use causes abnormalities in brain regions involved in sensory and emotional regulation. However, with long-term discontinuation and isolation, abnormalities in these brain regions can be partially reversed. Consistent with the current study, previous studies have reported that recovery of cognitive function and mood impairment occurs in methamphetamine users who undergo detoxification (Bensmann et al., 2019; Proebstl et al., 2019; Liu et al., 2021), and a gradual convergence of gray matter volume to normal size in the amygdala and parts of the temporal and occipital lobes of methamphetamine users after a period of discontinuation (Morales et al., 2012; London et al., 2015; Nie et al., 2020). However, due to the lack of an acute methamphetamine user group for comparison, our results should be interpreted with caution.

The present study has some limitations. First, the results of the current study were not corrected by stricter corrections, such as false discovery rate (FDR) or family-wise error (FWE), but by a more liberal scale of multiple comparison correction (GRF-corrected, *p* < 0.05). Considering that the recruited group included individuals with long-term methamphetamine withdrawal, we chose a more lenient multiple comparison correction. The correlation analysis results also supported this option, as the anomalous amplitude of the detected FC was negatively correlated with withdrawal duration. Although lenient corrections for multiple comparisons are more likely to detect residual abnormalities in long-term methamphetamine abstainers, these findings should be interpreted with caution, and further large-sample studies are needed to validate the results. Second, this was a case-control study that lacked follow-up data, and more longitudinal studies are needed to elucidate the relationship between the presence of sensory system abnormalities and methamphetamine use. Third, this study only recruited Chinese men. Women and long-term methamphetamine abstainers of other ethnicities should be studied as well. This study used a larger and more representative sample size. Finally, there were two limitations of this study to evaluate participants’ emotional states. One is we solely asked the participants about their current level of fear and the specific object of their fear (mainly focusing on claustrophobia). However, we did not gather additional information to assess participants’ fear using scales or interviews. Another one is that although we excluded participants with PHQ-9 scores above 5 (potentially depressed abstainers and healthy controls), we did not record the PHQ-9 scores of those included participants. These limitations hindered the further examination of whether changes in FC were influenced by mood states acting as confounding factors. Future studies should include the fear assessment scale and record depression scores for correlation analysis and comprehensive research.

## 5 Conclusion

This study explored amygdala-based resting-state FC in long-term methamphetamine abstainers at whole-brain voxel-wise. We observed that compared with HCs, long-term methamphetamine abstainers showed significant hyperconnectivity between the left lateral amygdala and parts of the temporal and occipital gyri. This hyperconnectivity was highly negatively correlated with withdrawal duration. Therefore, we speculate that the use of methamphetamine can impair the neural sensory system, including the visual and auditory systems, but this abnormal connectivity can gradually and partially recover after prolonged withdrawal of methamphetamine. Currently, effective methods for treating methamphetamine addiction are lacking. Nevertheless, our research suggests that withdrawal therapy may contribute to the restoration of sensory impairments in methamphetamine users. Despite this, owing to certain limitations inherent to our study, a study with a larger sample size could further explore the comprehensive impact of withdrawal therapy on individuals who use methamphetamine.

## Data Availability

The raw data supporting the conclusion of this article will be made available by the authors, without undue reservation.
