# Supplementary figures and images for: Hyperconnectivity of the lateral amygdala in long-term methamphetamine abstainers negatively correlated with withdrawal duration
Source: Front Pharmacol. 2023 Nov 10;14:1138704. doi: 10.3389/fphar.2023.1138704 (PMC10668120; doi:10.3389/fphar.2023.1138704)

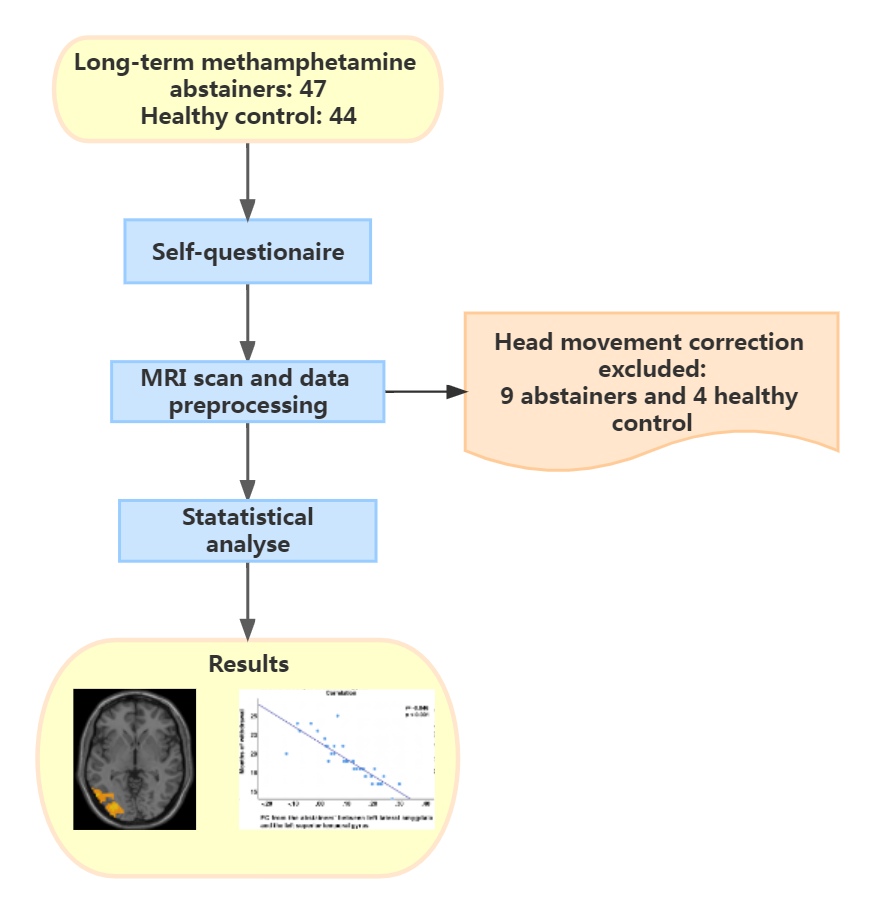

Supplement: Supplementary file 1 [file Image1.JPEG]
